# Challenges and advantages in wide-field optical coherence tomography angiography imaging of the human retinal and choroidal vasculature at 1.7-MHz A-scan rate

**DOI:** 10.1117/1.JBO.22.10.106018

**Published:** 2017-10-31

**Authors:** Raju Poddar, Justin V. Migacz, Daniel M. Schwartz, John S. Werner, Iwona Gorczynska

**Affiliations:** a University of California Davis, Vision Science and Advanced Retinal Imaging Laboratory (VSRI), Department of Ophthalmology and Vision Science, Sacramento, California, United States; b Birla Institute of Technology, Department of Bio-Engineering, Mesra, Ranchi, Jharkhand, India; c University of California San Francisco, Department of Ophthalmology and Vision Science, San Francisco, California, United States; d Nicolaus Copernicus University, Institute of Physics, Toruń, Poland

**Keywords:** optical coherence tomography, retinal blood flow, choroidal blood flow, ophthalmic optics and devices, ophthalmology, medical and biological imaging, optical coherence tomographyangiography, swept-source optical coherence tomography, Fourier-domain mode-locked laser

## Abstract

We present noninvasive, three-dimensional, depth-resolved imaging of human retinal and choroidal blood circulation with a swept-source optical coherence tomography (OCT) system at 1065-nm center wavelength. Motion contrast OCT imaging was performed with the phase-variance OCT angiography method. A Fourier-domain mode-locked light source was used to enable an imaging rate of 1.7 MHz. We experimentally demonstrate the challenges and advantages of wide-field OCT angiography (OCTA). In the discussion, we consider acquisition time, scanning area, scanning density, and their influence on visualization of selected features of the retinal and choroidal vascular networks. The OCTA imaging was performed with a field of view of 16 deg (5  mm×5  mm) and 30 deg (9  mm×9  mm). Data were presented in *en face* projections generated from single volumes and in *en face* projection mosaics generated from up to 4 datasets. OCTA imaging at 1.7 MHz A-scan rate was compared with results obtained from a commercial OCTA instrument and with conventional ophthalmic diagnostic methods: fundus photography, fluorescein, and indocyanine green angiography. Comparison of images obtained from all methods is demonstrated using the same eye of a healthy volunteer. For example, imaging of retinal pathology is presented in three cases of advanced age-related macular degeneration.

## 1 Introduction

The posterior segment of the human eye contains two distinct vascular networks: retinal and choroidal. The retinal vasculature is confined to the inner layers of the retina, from the nerve fiber layer to the outer plexiform layer, and is responsible for blood delivery to and drainage from those layers. The choroidal vasculature is located posterior to the retinal pigment epithelium (RPE) and is isolated from the retina by Bruch’s membrane. The function of the choroidal circulation is to provide nutrients to the photoreceptor layer, remove waste materials from the visual cycle, and thermoregulation. Investigation into vascular systems of the eye fundus is important in studies on normal eye function and development of diseases. Many of the ocular diseases leading to blindness, including diabetic retinopathy,^1^ central serous chorioretinopathy,^2^ age-related macular degeneration (AMD),^3^ and glaucoma,^4^ have a vascular component, which manifests as changes in the blood circulation and changes in the vascular morphology. The conventional diagnostic methods used to assess the vascular component of disease development include: fundus photography (FP), fluorescein angiography (FA), and indocyanine green angiography (ICGA). The typical field of view (FOV) used in these techniques is 30 deg to 45 deg, but wide-field fundus cameras can provide an FOV of ∼60  deg.^5^^–^^7^ Recently, ultrawide-field imaging systems enabling ∼200-deg FOV have been developed for FP, FA, and ICGA imaging.^5^^,^^6^

The advantage of these clinical angiography imaging techniques is that they provide a fast, motion artifact-free, image of the retina. However, they can provide essentially no depth information. FA and ICGA can provide time-resolved analysis of the blood flow distribution. Yet, injection of fluorescent dyes into the blood stream is required, which poses a risk of adverse reactions. Optical coherence tomography angiography (OCTA) imaging techniques can overcome these drawbacks of the classic methods. However, one of the limitations of OCTA is relatively slow imaging speed, making it difficult to capture a wide FOV.

In spectral-domain OCT (SDOCT), the imaging speed is limited by the cameras used in the spectrometers to acquire the OCT signal. Commercially available SDOCT devices operate at ∼70-kHz A-scan rate. Systems developed in research laboratories can operate at imaging speeds of ∼100 to 300 kHz with a single camera used for the detection.^8^ The imaging speed can be further increased to over ∼0.5  MHz with the use of more than one spectrometer.^9^^,^^10^

In swept-source OCT (SSOCT) devices, the imaging speed is limited by the sweep rate of the light source. In the commercially available ophthalmic SSOCT systems, the maximum sweep rate is 100 kHz. Research grade systems developed for the imaging of the posterior segment of the eye can operate at imaging speeds of 100∼400  kHz with the use of short cavity swept source lasers.^11^^–^^18^ Imaging speeds beyond 1 MHz can be achieved with Fourier-domain mode-locked (FDML) lasers.^18^^,^^19^ Another technique enabling over 1-MHz imaging is full-field swept source OCT.^20^ Advances in high-speed OCT imaging techniques enabled development of OCT velocimetry (OCT-V) and OCTA methods. OCT-V methods utilize a variety of phenomena to assess some, or all components of the flow velocity vector. These phenomena include: Doppler effect,^21^ Doppler OCT signal broadening,^22^^,^^23^ and complex OCT signal decorrelation rate^24^ (measured for example, as complex amplitude, speckle intensity decorrelation or variance, phase decorrelation or variance, or combinations thereof). OCTA^25^ methods use the same phenomena; however, they are not utilized to quantify the flow but are used as intrinsic contrast mechanisms for vascular morphology imaging and quantification.^26^^,^^27^

In recent years, many OCTA methods have been developed for the imaging of retinal and choroidal vasculature, including: speckle-variance OCTA,^28^ phase-variance OCTA,^29^ amplitude-decorrelation OCTA (including the split spectrum variant: SSADA),^30^ correlation mapping OCA,^31^ optical angiography,^32^ optical microangiography (OMAG),^33^ and power Doppler.^34^^,^^35^

Unlike classic, fundus photography, or scanning laser ophthalmosocopy (SLO)-based angiography methods, OCTA techniques provide depth information and enable isolation of all vascular retinal^36^^,^^37^ and selected choroidal^27^^,^^38^^–^^41^ layers. However, imaging speed limitations of the OCT techniques require compromises among a set of imaging parameters. In clinical diagnostics, there is a demand for (1) large FOV, (2) short imaging time, and (3) highly detailed image features. An FOV similar to a standard fundus photograph (∼30  deg to 45 deg) is usually preferred by ophthalmologists to aid in the diagnosis of retinal diseases, but this cannot be achieved with current OCT imaging speeds at high resolution. The rule of thumb for the OCT imaging time is ≤5  s to reduce motion artifacts in the images and to avoid image quality degradation caused by the drying of the tear film. Image quality depends on both the beam spot diameter at the retina and scanning step size. Current speeds of typical OCT devices do not fulfill all three needs for clinical OCTA imaging. If a typical OCT system (100-kHz A-scan rate, 14-μm transverse imaging resolution) is used for OCTA imaging, in which four B-scans are used for the flow metric calculation, then scanning an FOV of 30 deg (9×9  mm) with a step size of 7×7  μm will result in a dataset consisting of 5140 B-scans and 1285 A-scans each. Acquisition time of this dataset would take 1.1 min, which is far beyond acceptable imaging times. If OCTA imaging with an FOV similar to a wide-field fundus angiography is required (60 deg, 18×18  mm), the data size would be 10284×2571 A-scans, and the acquisition would take 4.4 min. Clearly, compromise between FOV, scanning step, and imaging time is required in OCTA imaging, depending on specific diagnostic demands. Such compromises have been proposed in previous reports. In the majority of these cases, extending the FOV beyond 10 deg (3 mm) without exceeding the imaging time beyond 5 to 7 s was achieved by undersampling in at least one scanning direction.^42^^,^^43^ Some authors report reduction in the number of B-scans used for OCTA computations.^44^^,^^45^

However, these two approaches may compromise visualization of finer vascular features. In addition, none of these papers reported imaging with an FOV comparable to the classic wide-field fundus angiography (∼60  deg) in a single dataset. One strategy for achieving a wider FOV in OCTA imaging requires creation of mosaics of several narrow-field images acquired in adjacent locations in the eye fundus. However, this prolongs the patient’s imaging session and requires development of data processing methods for overlapping and merging the mosaic tiles.^46^ Some groups have reported wide-field OCTA with real-time motion tracking with a line scan ophthalmoscope for real-time stitching of narrow-field images.^42^ Wide-field OCTA imaging in a single dataset was for the first time reported after the advent of FDML lasers operating at 1060-nm center wavelength and 1.7-MHz imaging speed.^38^

In this paper, we demonstrate OCTA imaging of the posterior eye segment vasculature with an FDML laser operating at 1.7-MHz sweep rate, at 1065-nm center wavelength. The phase-variance method was used for OCT angiography imaging. Images of the retinal and choroidal vasculature with an FOV of 16 deg and 30 deg were captured in single three-dimensional (3-D) datasets. We compare the possibility of imaging selected vascular features depending on the two scan densities. We discuss trade-offs and challenges connected to the imaging speed in the wide-field OCTA, considering acquisition time, scanned area, and scanning density. Imaging was performed in the eyes of a normal subject and three patients diagnosed with AMD.

## 2 Materials and Methods

### 2.1 Study Subjects

Imaging of human subjects was performed under a protocol approved by the UC Davis Institutional Review Board and was in accordance with the declaration of Helsinki. Eyes of a healthy volunteer, 51-year-old Caucasian female (N1), and three patients diagnosed with advanced AMD: geographic atrophy (GA), P1 and P3 ages 60, 81, choroidal neovascularization treated with anti-VEGF injections, P2, age 67, was imaged with a swept-source OCT (SSOCT) system. Subject preparation included instillation of eye drops: 1% tropicamide and 2.5% phenylephrine, for pupil dilation and cycloplegia. Head position was stabilized with a forehead rest and a bite bar. A white fixation mark was displayed on a liquid crystal display to guide the subject’s gaze to select retinal locations for OCT imaging. The light power at the cornea was 1.8 mW, which is below the ANSI limits for safe eye exposure to near-infrared light centered at 1065 nm.^47^

### 2.2 Clinical Ophthalmic Imaging

Standard ophthalmic imaging was performed to enable comparison of features imaged with the 1.7-MHz sweep rate OCTA system and findings visualized using classic diagnostics methods. Color fundus photographs and FA images were taken with a Topcon (TRC-50IX) camera, and ICGA images were collected with the Spectralis HRA + OCT system. In addition, imaging was performed with AngioPlex Cirrus HD-OCT Angiography apparatus (Carl Zeiss Meditec, Inc., California, USA) a spectral-domain OCT (SDOCT) system with 840-nm center wavelength.^45^ The imaging with 5-μm axial resolution at full width at half maximum (FWHM), and 15-μm $1/e^{2}$ beam spot diameter at the retina was performed at 68-kHz A-scan rate. The scanning protocols and the parameters of vascular layer visualization are presented in Tables 1 and 2. The OCTA method implemented in the AngioPlex Cirrus HD-OCT instrument is OMAG. All images from the latter system were generated by the AngioPlex software.

**Table 1:** Scanning protocols in the Zeiss AngioPlex apparatus.^45^^,^^48^

Parameter	AngioPlex	AngioPlex
	protocol:1	protocol: 2
Imaged area	3 mm×3 mm	6 mm×6 mm
FOV	10 deg	20 deg
Pixels per A-scan	1024	1024
Number of A-scans per B-scan	245	350
Step size (isotropic in horizontal and vertical direction)	12 μm	17 μm
Beam spot diameter at the retina	15 μm	15 μm
B-scan repeats (B-scans per MB-scan)	4	2
Number of BM-scans (slow scanner positions)	245	350
Number of B-scans per volume	980	700
B-scan repetition time	3.8 ms	5 ms
Volume acquisition time	4 s	4 s

**Table 2:** Axial windowing parameters for *en face* projection imaging in Zeiss AngioPlex sdOCT apparatus.

Imaged vascular layer	Axial span of OCTA en face projections
Superficial retina	From the inner limiting membrane to the outer boundary of the inner plexiform layer, (layer segmentation was performed with the Zeiss AngioPlex software).
Choriocapillaris	From ∼30- to 50-μm posterior to the RPE peak.
Choroid	From ∼65- to 115-μm posterior to the RPE peak.

### 2.3 FDML Swept-Source OCT System

**Fig. 1:**
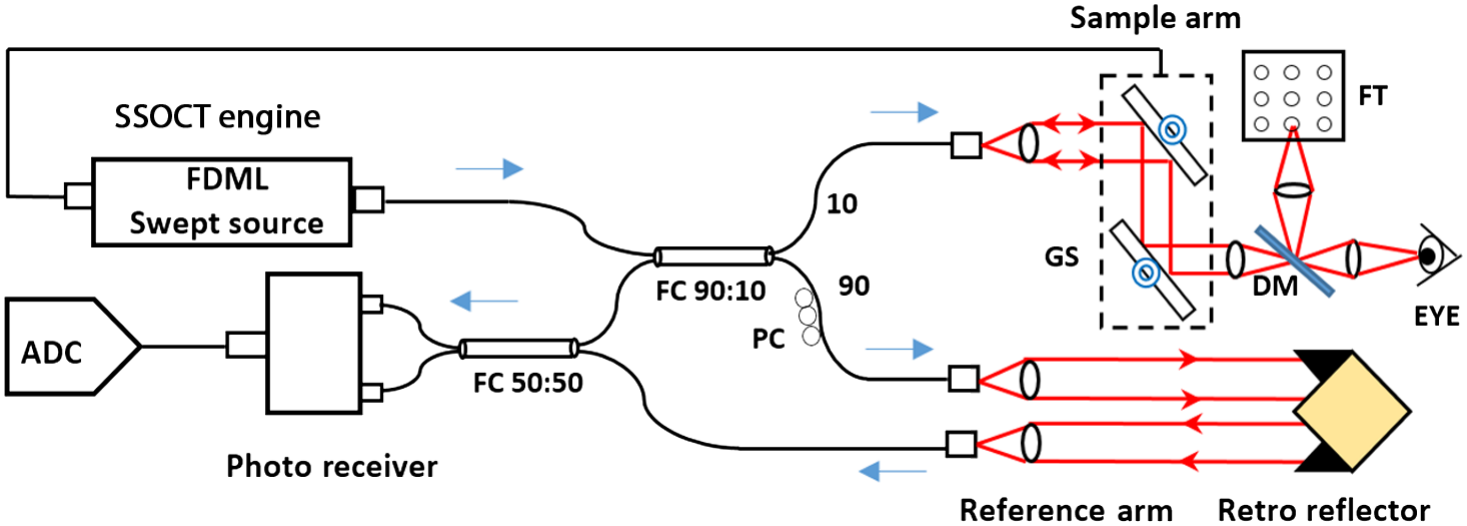
A diagram of the FDML SSOCT system. FC, fiber coupler; GS, galvanometer; OS, optical scanners; PC, polarization controller; DM, dichroic mirror; FT, fixation target; and ADC, digitizer.

Imaging was performed with a swept-source OCT system (Fig. 1 and Table 3). A Fourier-domain mode-locked laser (FDML-1060-750-4B-APC, OptoRes GmbH, Germany) was used as the light source with wavelength-sweep frequency of 1.7 MHz, enabling OCT imaging at 1.7 million A-scans/s. The center wavelength of the emitted light was 1065 nm with 80-nm spectral bandwidth, providing axial imaging resolution of 7  μm (FWHM) in tissue. The depth imaging range was 3.5 mm in tissue, and the peak sensitivity was 92 dB. The imaging platform was designed to provide a maximum FOV of 40 deg. A 90:10 fiber coupler (Gooch & Housego Ltd., UK) was used in the Mach–Zehnder interferometer to split the light between the object arm (10%) and the reference arm (90%).The object arm was constructed using

• collimator: two achromatic lenses (Thorlabs, New Jersey, USA), f=19  mm each, effective focal length ∼11  mm,
• galvanometer scanners (Cambridge Technology, Massachusetts, USA), 3-mm clear aperture,
• scan lens: two achromats (Thorlabs, USA), f=100  mm each, effective focal length ∼50  mm,
• a dichroic mirror (Chroma Technology, Vermont, USA): transmission of near-infrared light and reflection of visible light, used for coupling of the eye fixation channel with the OCT imaging channel, and
• ocular lens: two achromats (Thorlabs, USA), f=80  mm each, effective focal length ∼40  mm.

**Table 3:** Specifications of the FMDL swept-source OCT system.

Parameter	Value
FDML laser sweep rate	1.7 MHz
FDML laser sweep (A-scan) acquisition time	0.584 μs
Central wavelength	1065 nm
Optical bandwidth (FWHM)	80 nm
Axial imaging resolution (in tissue, n≈1.33)	7 μm
$1/e^{2}$ beam spot diameter at the retina	14 μm
Axial imaging range	3.5 mm
Light power at the cornea	1.8 mW
Peak imaging sensitivity	92 dB

The reference arm consisted of a collimating lens (f=10  mm, Thorlabs, USA), an iris with adjustable aperture (for controlling the light power), a cube retro-reflector (Thorlabs, USA), and a light-collecting lens (f=10  mm, Thorlabs, USA). The light dispersion in the excess of glass in the optical elements of the object arm was compensated with an optical fiber of an experimentally matched length. The polarization control was achieved by a three-paddle polarization controller (Thorlabs, USA). The OCT signal was acquired with a dual-balanced detection system containing a 50:50 fiber coupler (Gooch & Housego Ltd., UK) and a pair of photodiodes (Thorlabs, USA), and digitized using a 12-bit digitizer with sampling speed of 1.8 billion samples/s (ATS9360, Alazar Tech, Canada).

### 2.4 OCT Angiography Scanning Protocols Used in FDML SSOCT System

An overview of the OCT angiography scan protocols used in our FDML SSOCT system is shown in Table 4. Two raster scan protocols with isotropic scanning densities were used: one with a 5-μm scan step, and one with an 8-μm scan step. The scanning step in FDML SSOCT protocol 1 was smaller than half of the $1/e^{2}$ beam spot diameter at the retina (14  μm), while the scanning step in FDML SSOCT protocol 2 exceeded that value. Five B-scans were acquired at each position of the slow scanner (per each BM-scan) to enable OCT angiography imaging. A large number of over 1000 A-scans per B-scan and BM-scans per volume were acquired to image the largest possible area of the retina in a time of 6 to 8 s. The data sizes were 11 and 14 GB.

**Table 4:** FDML SSOCT angiography scan protocols.

Parameter	FDML	FDML
	SSOCT	SSOCT
	Protocol: 1	protocol: 2
Imaged area	5 mm×5 mm	9 mm×9 mm
FOV	16 deg	30 deg
Pixels per A-scan	1024	1024
Number of A-scans per B-scan	1024	1152
Step size (horizontal and vertical)	5 μm	8 μm
$1/e^{2}$ beam spot diameter at the retina	14 μm	14 μm
B-scan repeats (B-scans per BM-scan)	5	5
Number of BM-scans (slow scanner positions)	1024	1152
Number of B-scans per volume	5120	5760
B-scan repetition time (including fly-back)	1.2 ms	1.35 ms
Volume acquisition time	6 s	8 s
Data size	11 GB	14 GB

### 2.5 Data Processing

Data processing consisted of three steps: (i) processing of the acquired spectral interference signals, (ii) OCT angiography computations, and (iii) implementation of 3-D data visualization methods for *en face* projection imaging.

Numerical processing of the OCT signal included generation of the spectral interference pattern in wave number space, from the signal acquired in the wavelength domain, with the use of calibration fringe pattern (often called a k-clock) acquired prior to the patient imaging sessions. Fixed pattern noise was estimated by averaging of all acquired interference spectra, and removed by subtracting the computed mean interference spectrum from each B-scan. Numerical compensation of the residual dispersion mismatch of light propagating in the reference and object arms of the interferometer was performed to avoid axial imaging resolution degradation. Spectral shaping was implemented to reduce image artifacts introduced by the side lobes of the coherence function (OCT axial point-spread function). Finally, digital Fourier transformations with zero-padding to 4096 points per spectrum was performed yielding complex amplitudes corresponding to OCT A-scans.

For angiogram generation, we have selected the phase-variance method (PV OCTA).^29^ Magnitudes and phases of the interference fringe pattern at each imaged depth were extracted from the complex amplitudes. The magnitudes were used for intensity thresholding performed to exclude noise pixels from phase-variance computations, and for generation of mean OCT intensity cross-sectional images revealing the tissue morphology. Mean B-scans were computed by averaging B-scans within BM-scans. The phases were used to generate cross-sectional OCTA images of the vascular morphology. The OCTA computations included: removal of the phase shifts caused by bulk eye motion (a shifted histogram analysis method was used^25^), and computation of phase-variances among B-scans within BM-scans. High values of the phase-variance indicate the location of the moving blood cells within the tissue volume. Projections of the blood flow in the retinal vessels onto deeper layers (blood flow-projection^49^ artifacts^37^) were not removed.

**Fig. 2:**
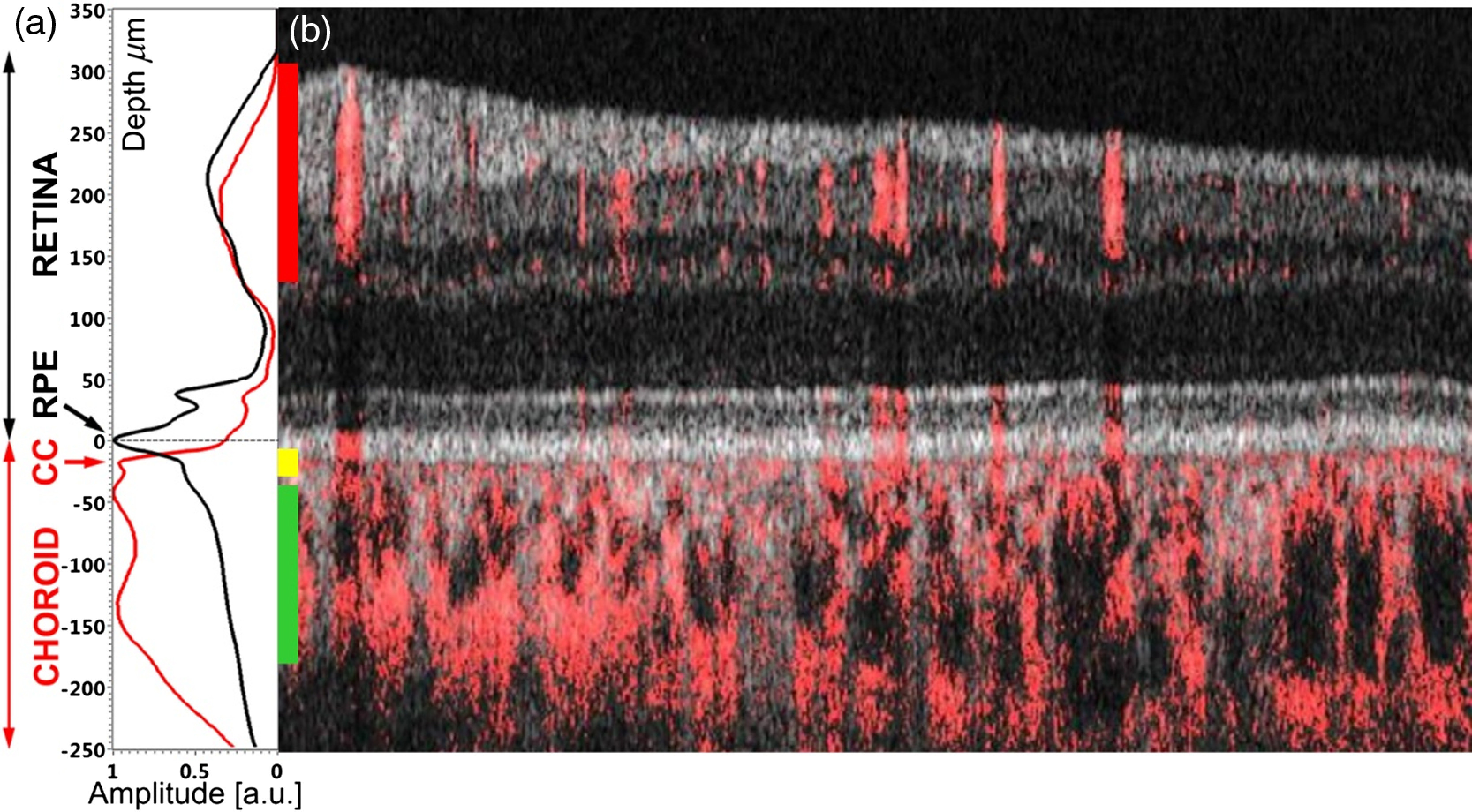
Example result of cross-sectional images flattened to the RPE obtained in a healthy subject (N1). (a) Mean axial PV OCTA profile (red line) and intensity profile (black line) created by summation of all OCTA and OCT A-scans within the 3-D dataset, CC, choriocapillaris; RPE, retinal pigment epithelium. (b) A composite cross-sectional image generated by an overlay of PV OCTA B-scan (red) with an intensity B-scan (grayscale). Vertical bars in (b) indicate locations and thicknesses of layers selected for the visualization of retinal (red bar), and choroidal (yellow and green bars) vasculature. Horizontal image size: 5 mm.

Flattening of the B-scans and OCTA cross-sections was performed using methods described in a previous paper.^36^ The image processing included: segmentation of the RPE in the intensity B-scans, flattening the cross-sectional images to the RPE [Fig. 2(b)] using Zernike polynomial fits, removal of the bulk motion affected B-scans from the 3-D datasets. *En face* projections were generated with ImageJ software^50^ by axial summation of the data within square windows at selected depth locations relative to the RPE position (Table 5). To remove the residual noise from the *en face* OCT angiography images, median filtering was applied followed by a Gaussian filter. Image filtering was performed with ImageJ.

**Table 5:** Axial windowing parameters for *en face* projection imaging in FDML SSOCT system.

Imaged vascular layer	Window location	Window width
Joint retinal layers	∼90 μm, anterior to RPE peak	∼138 μm
Choroid 1 (CHRD1)	∼12 μm, posterior to RPE peak	∼28 to 39 μm
Choroid 2 (CHRD2)	∼127 μm, posterior to RPE peak	∼120 to 180 μm

## 3 Results

### 3.1 Influence of Scan Density on OCTA Imaging

**Fig. 3:**
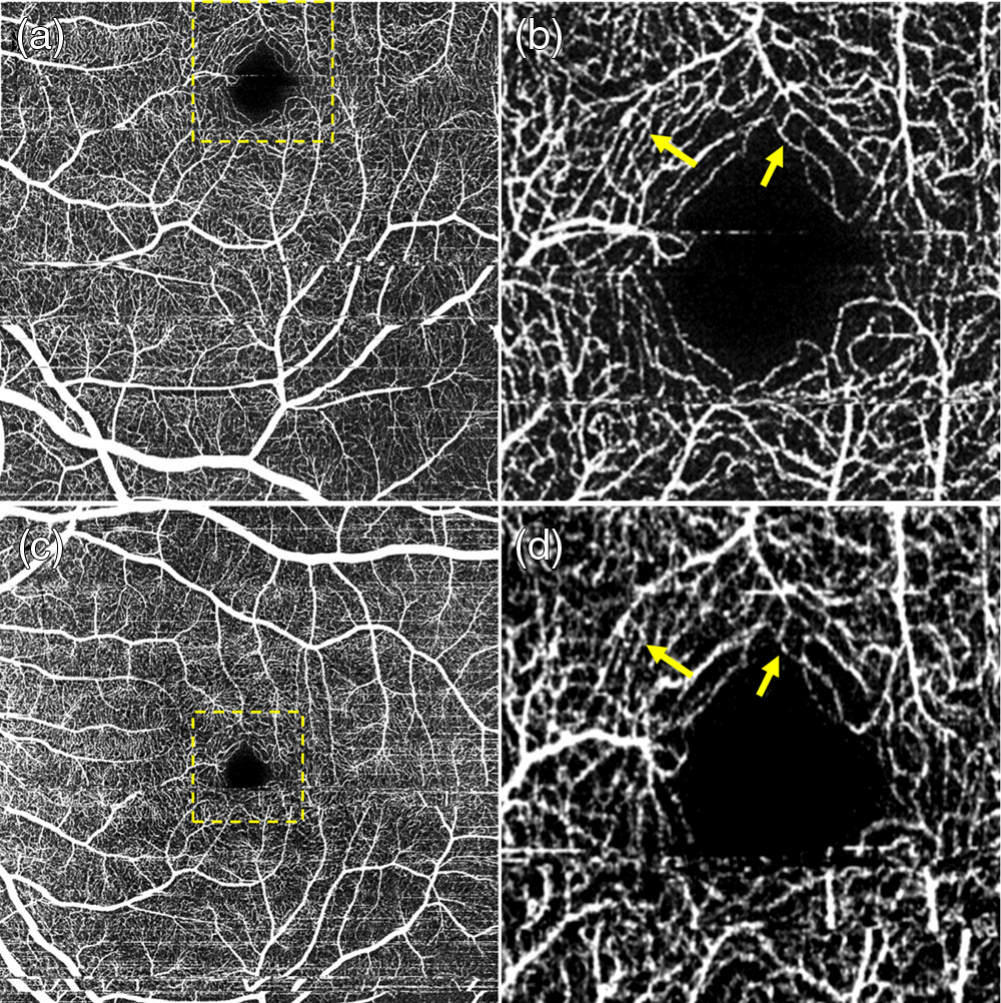
FDML SSOCT at 1.7-MHz A-scan rate, PV OCTA *en face* projections of the retinal vasculature. Normal subject, age 51. Top row: scanning step of 5  μm. Bottom row: scanning step of 8  μm. Left column: full imaged area. Right column: zoom at 1.5  mm×1.5  mm area centered at the fovea. (a) 1024×1024  pixels, 5-mm×5-mm area imaged in 6 s. (c) 1152×1152  pixels, 9-mm×9-mm area imaged in 8 s. Depth positions and ranges used for generating the *en face* images are given in Table 3. Dashed yellow square shows the zoomed image portion. (b) Arrows indicate example vessels with more continuous appearance and (d) less clearly defined.

**Fig. 4:**
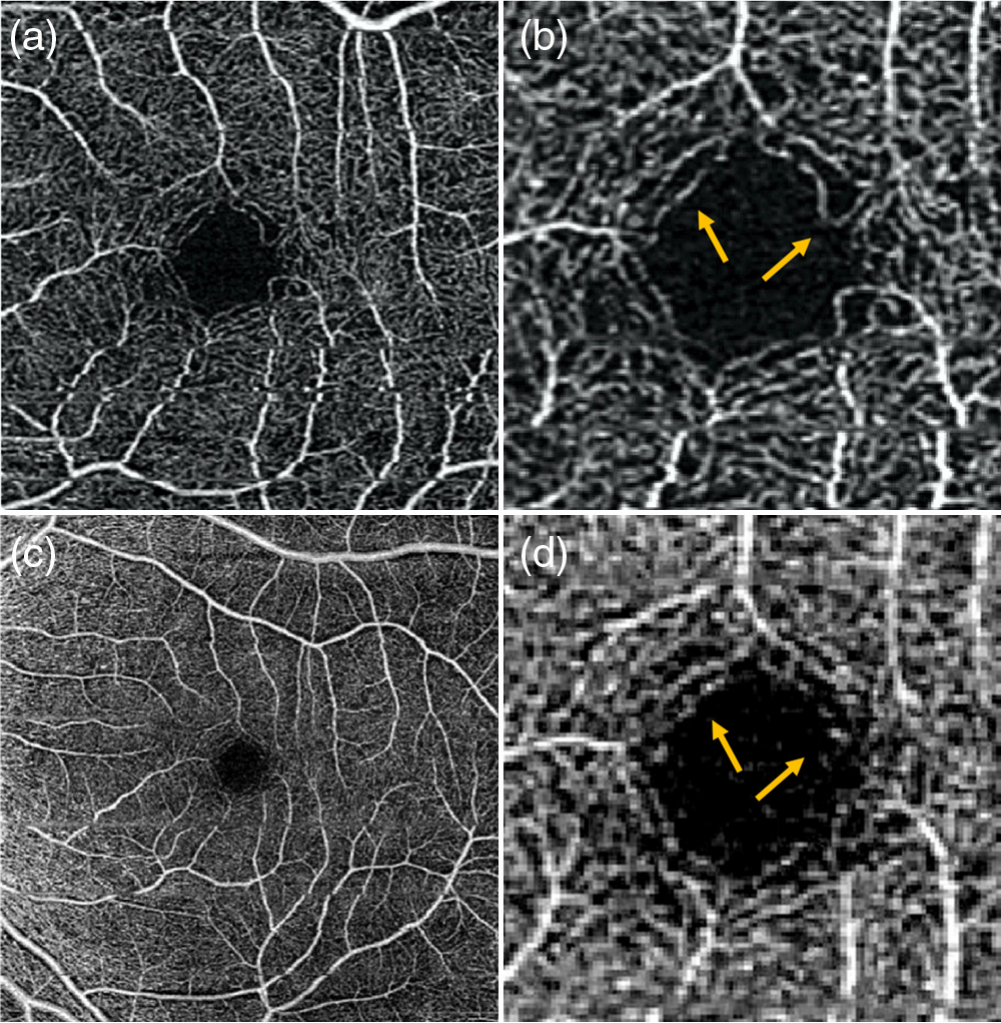
Zeiss Angioplex 68-kHz A-scan rate, OCTA *en face* projections of the superficial retina. Normal subject (N1), age 51. Top row: scanning step 12  μm. Bottom row: scanning step 17  μm. Left row: full imaged area. Left row: zoom at 1.5-mm×1.5-mm area centered at the fovea. Image sizes: (a) 3  mm×3  mm, (c) 6  mm×6  mm, and (b and d) 1.5  mm×1.5  mm.

**Fig. 5:**
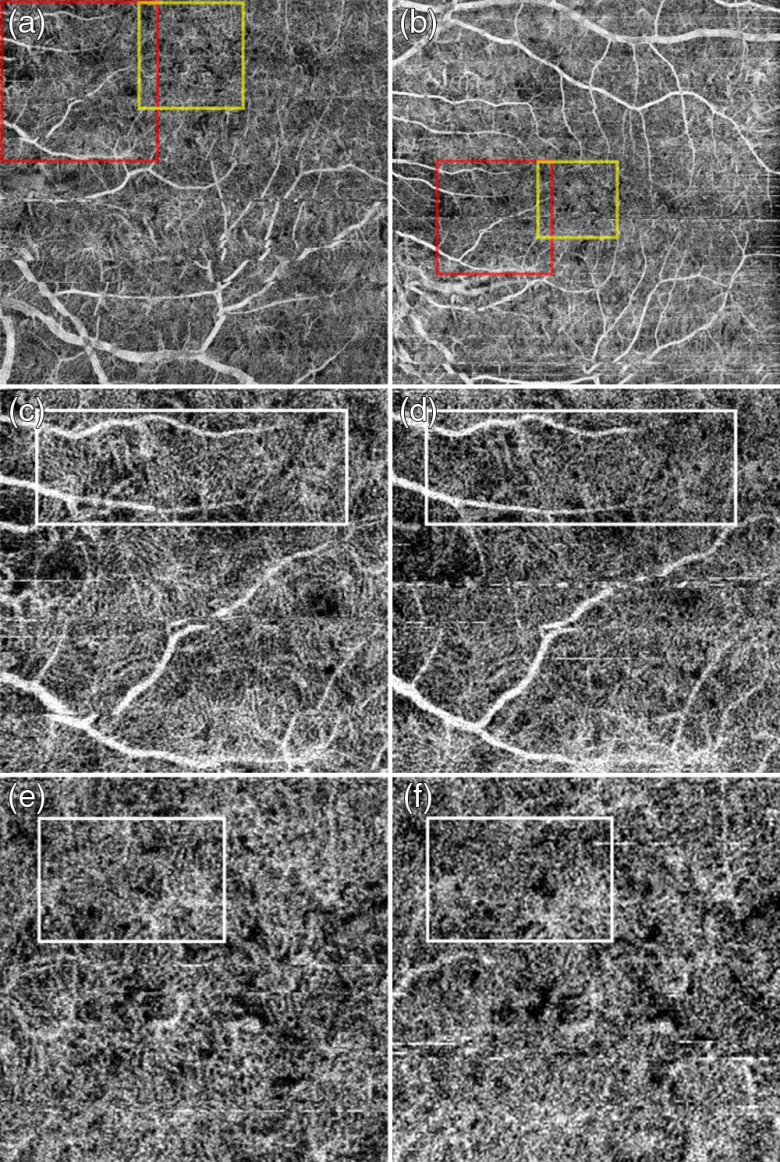
FDML SSOCT at 1.7-MHz A-scan rate, PV OCTA *en face* projections of the choriocapillaris. Normal subject, age 51. Left column: scanning step 5  μm. Right column: scanning step 8  μm. Top row: full imaged area. Middle row: zoom at 2-mm×2-mm area indicated by red square in (a) and (b). Bottom row: zoom at 1.5-mm×1.5-mm area centered at the fovea as indicated by yellow square in (a) and (b). Image sizes: (a) 5  mm×5  mm, (b) 9  mm×9  mm, (c and d) 2  mm×2  mm, and (b and d) 1.5  mm×1.5  mm. Depth position and range selected for generation of the *en face* images are given in Table 3. (c and e) White rectangles indicate region of interest with appearance resembling the meshwork of choriocapillaris vessels and (d and f) less clear visualization of the meshwork due to higher pixilation noise.

**Fig. 6:**
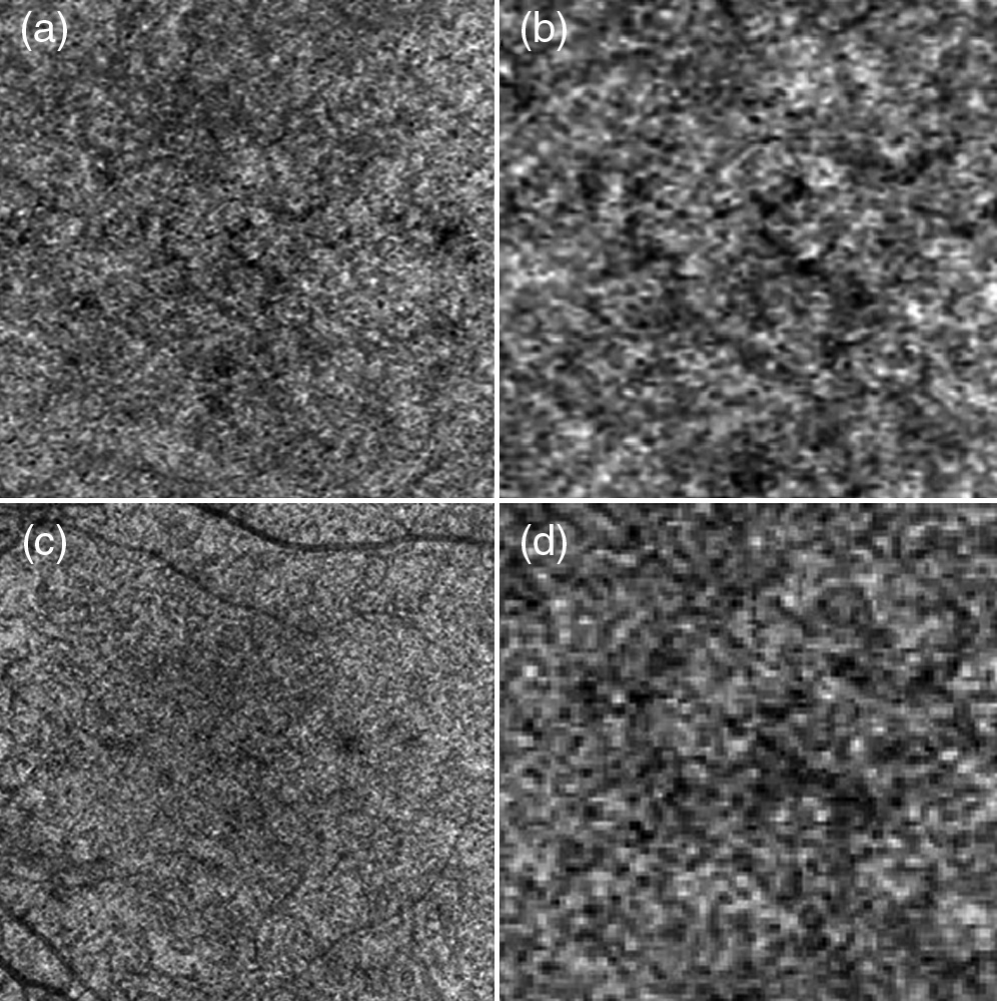
Zeiss Angioplex, 68-kHz A-scan rate, OCTA *en face* projections of the choriocapillaris. Normal subject, age 51. Top row: scanning step 12  μm. Bottom row: scanning step 17  μm. Left row: full imaged area. Left row: zoom at 1.5-mm×1.5-mm area centered at the fovea. Image sizes: (a) 3  mm×3  mm, (c) 6  mm×6  mm, and (b and d) $1.5\;\mathrm{mm}\times 1.5\;{\mathrm{mm}}^{2}$.

**Fig. 7:**
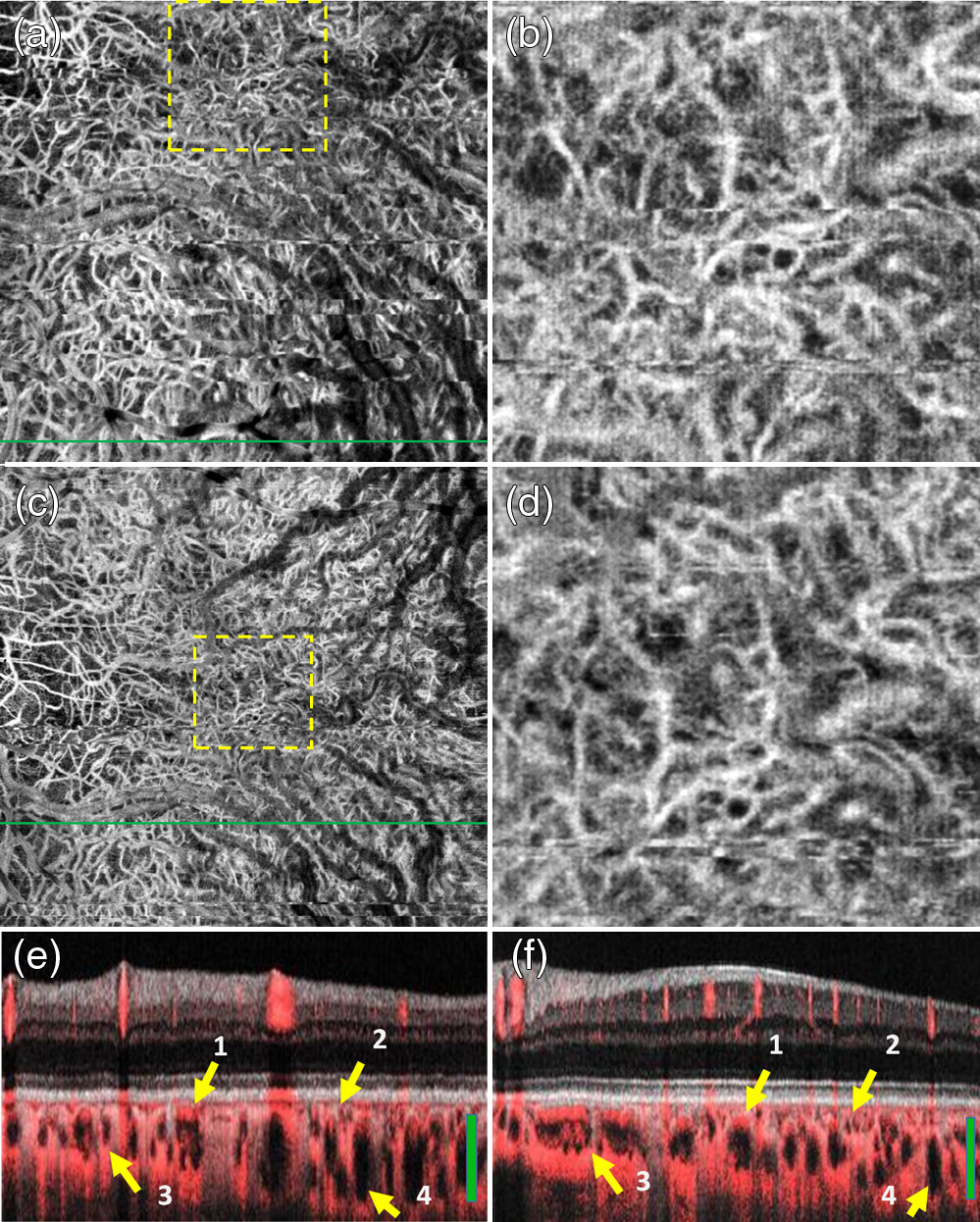
FDML SSOCT at 1.7-MHz A-scan rate, PV OCTA *en face* projections of the choroid. Normal subject, age 51. Left column: scanning step 5  μm. Right column: scanning step 8  μm. Top row: full imaged area. Middle row: zoom at 1.5-mm×1.5-mm area centered at the fovea. Bottom row: overlay of OCTA images (red) and intensity B-scans (gray scale); arrows indicate example: 1—large vessels with OCTA signal visible in the top part of the vessel, 2—small vessels with OCTA signal visible across the lumen, 3—vessels appearing as white in the *en face* projection due to inclusion of flow projections, and 4—vessels appearing as dark; green bars indicate position of the *en face* projections. Green line in *en face* angiograms (a) and (c) showing the location of the B-scans (e) and (f), respectively. Image sizes: (a) 5  mm×5  mm, (b) 9  mm×9  mm, and (c and d) 1.5  mm×1.5  mm.

**Fig. 8:**
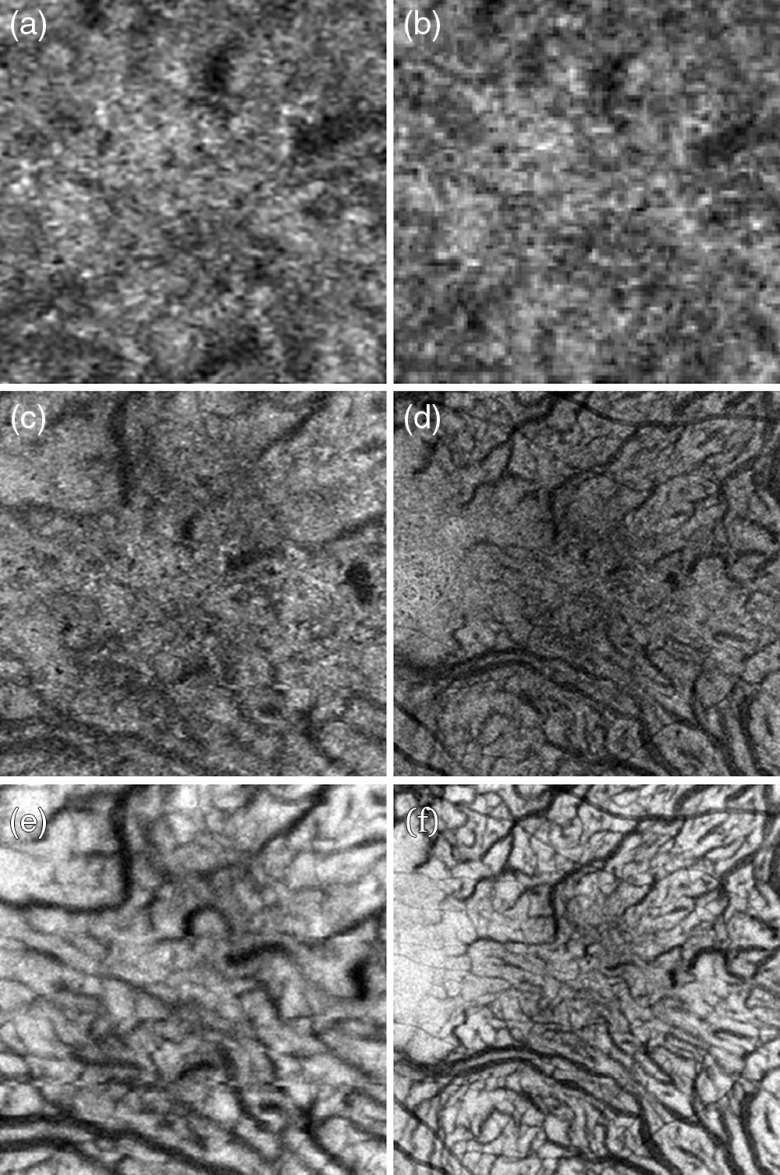
Zeiss Angioplex 68-kHz, A-scan rate, and OCTA and OCT intensity *en face* projections of the choroid. Normal subject, age 51. Left column: scanning step 12  μm. Right column: scanning step 17  μm. Top row: OCTA, zoom at 1.5-mm×1.5-mm area centered at the fovea. Middle row: OCTA, full imaged area. Bottom row: OCT intensity projections. Image sizes: (a and b) 1.5  mm×1.5  mm, (c and e) 3  mm×3  mm, and (d and f) 6  mm×6  mm.

The effects of scanning density on the OCT angiography visualization of vascular features were shown in Figs. 3–8. Figures 3, 5, and 7 present images obtained from the research-grade FDML SSOCT system. For comparison with current standards in clinical OCTA imaging, we have also provided angiograms generated with a commercial OCT angiography system (Figs. 4, 6, and 8). For consistency of the results (i.e., to avoid confounding factors caused by inter-subject variability), we have performed imaging using both systems in the same eye of a healthy volunteer (Caucasian female, age: 51 years).

OCT angiography imaging with the FDML SSOCT system provides visualization of not only the retinal (Fig. 3), but also choroidal vasculature (Figs. 5 and 7). Images obtained from the commercial OCT angiography apparatus provide images of the retinal vasculature (Fig. 4), but the details of the choroid are not visualized (Fig. 8). However, the reader should be made aware of the differences between the two imaging systems. The research instrument is a swept source OCT system operating at 1.7-MHz A-scan rate, with 1065-nm center wavelength of the light used for the imaging, and uses the PV OCT angiography method. The commercial apparatus uses the spectral-domain OCT technique, with a 68 kHz imaging rate and 840-nm center wavelength of the light emitted by a superluminescent diode and uses the OMAG OCTA method. The two OCT technologies, imaging speeds, and wavelength regimes may influence the possibility to image the choroid and detect the blood flow in the choroidal vessels.^51^

Therefore, the comparison of the images should focus on the details of the visualized vascular features depending on the scanning density. No attempt should be made to identify specific imaging parameters responsible for the lack of choroidal vasculature visualization in the commercial apparatus since our experiments were not designed to support such study.

Figure 3 shows *en face* projections of the retinal vasculature obtained from the FDML SSOCT system. Vessels arching around the fovea, and branching into vessels radiating toward/from the fovea are visible in images obtained from the 5- and 8-μm scan steps (0.36 and 0.57 of the $1/e^{2}$ beam spot diameter, respectively).

The image area between the large retinal vessels is occupied by a fine meshwork of capillary vessels, located between the inner and outer plexiform layers, or the intermediate and deep plexuses. Visualization of large retinal vessels is not influenced by the implemented scan densities [Figs. 3(a) and 3(c)]. Comparable levels of detail are also visible in the capillary network [Figs. 3(b) and 3(d)]. However, the apparent continuity of vessels in the areas free from saccadic motion artifacts is higher in the images obtained from higher scanning densities of the retinal vasculature, typical for OCTA imaging with commercially available instruments are shown in Fig. 4. Good visualization of large retinal vessels was obtained from the 12- and 17-μm scan steps (0.8 and 1.1 of the beam spot diameter, respectively). However, visualization of capillary vessels differs for the two scanning densities. While the 12-μm scan step provides visualization comparable with the FDML SSOCT system at lower scanning density, the 17-μm scan step causes loss of information. Discontinuities are visible in the ring of capillary vessels surrounding the foveal avascular zone [arrows in Figs. 4(b) and 4(d)]. Pixilation noise degrades the quality of the images.

*En face* projections of the “choriocapillais” layer generated with the FDML SSOCT system are presented in Fig. 5. The enlarged portions of the images obtained from the 5-μm scanning step size (less than half of the $1/e^{2}$ beam spot diameter) have an appearance resembling a meshwork of choriocapillaris. The meshwork is less evident in the images obtained from 8-μm scanning step due to higher pixilation noise. Example regions of interest minimally affected by motion artifacts are indicated by white rectangles. The images of deeper choroid (Fig. 7) show an extremely densely packed network of medium and large diameter vessels. As in OCTA imaging of the large retinal vessels, the visualization of large choroidal vessels is not affected by the two implemented scanning protocols. The appearance of the choroidal vessels in OCTA imaging depends on the size of the vessels and the blood flow velocity. In vessels with smaller lumen and slower flow, the OCTA signal is stronger, most likely due to less washout of the interference fringe pattern and due to less light scattering. These vessels appear white in the *en face* projections. In the vessels with faster blood flow and with larger lumen, the OCTA signal is weaker or indistinguishable from the noise making their appearance dark in the projection images. However, in most of the large vessels, the OCTA signal is still visible in their interior parts (on the top in the cross-sectional images). If the *en face* projection includes the top part of a large vessel, that vessel also appears white in the image. In addition, when the *en face* projection spans in depth below the vessels, the flow projections contribute to the OCTA signal enhancing the visualization.

The presence of the layer, but not the structure, of choriocapillaris was visualized with the commercial OCTA apparatus (Fig. 6). No OCTA signal was detected in deeper choroidal vessels (Fig. 8). Therefore, no reliable conclusions regarding influence of scan density on vascular imaging can be made. However, a few interesting features may be observed in the images of choriocapillaris and deep choroid. In Fig. 6, focal areas with no flow signal (black spots) are embedded within an apparently random distribution of the OCTA signal originating from unresolved choriocapillaris meshwork. The nonperfused spots probably correspond to larger gaps between the vessels (flow voids).^52^

In Fig. 8, large choroidal vessels are visible as areas of low OCTA signal embedded in high OCTA noise (pixels with random, high OCTA signal values), and appear similar to shadows of choroidal vessels observed in the OCT intensity projections. This suggests that even though blood flow signal cannot be detected in large choroidal vessels with the commercial apparatus, some information about the architecture of the choroidal vasculature can be retrieved in the form of such shadowgrams [Figs. 8(e) and 8(f)].

### 3.2 Comparison of High-Speed OCTA with FA and ICGA of a Normal Eye

To preserve imaging resolution, the spot diameter of the imaging beam should not exceed half of the $1/e^{2}$ beam diameter at the retina. Large scan steps cause loss of information about object features of high spatial frequencies (e.g., choriocapillaris), which could be obtained from higher scanning densities. This, however, compromises the capability of wide-field imaging with OCT angiography. Acquisition of data from only 35-deg FOV, considered as “standard” rather than “wide” in ophthalmic diagnostics, would take an unreasonably long time. A common solution to this challenge is breaking the OCTA imaging into acquisitions of data from smaller areas in neighboring locations in the object, and numerically “stitching” the resultant images into mosaics.

**Fig. 9:**
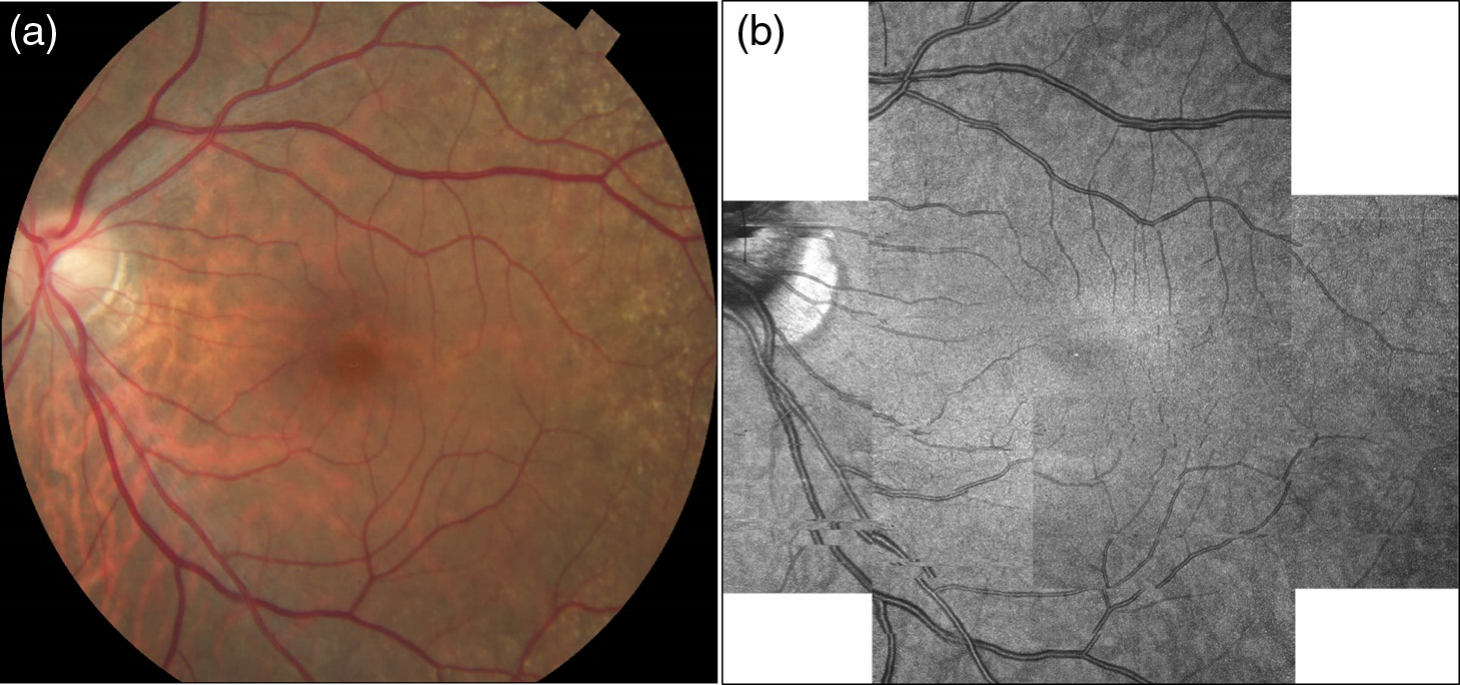
Comparison of fundus photograph (30-deg FOV) with OCT intensity full-depth projection, 30-deg mosaic composed of four $5\text{-}{\mathrm{mm}}^{2}$ OCT images. Images obtained in the volunteer N1—Caucasian female, 51-years-old.

**Fig. 10:**
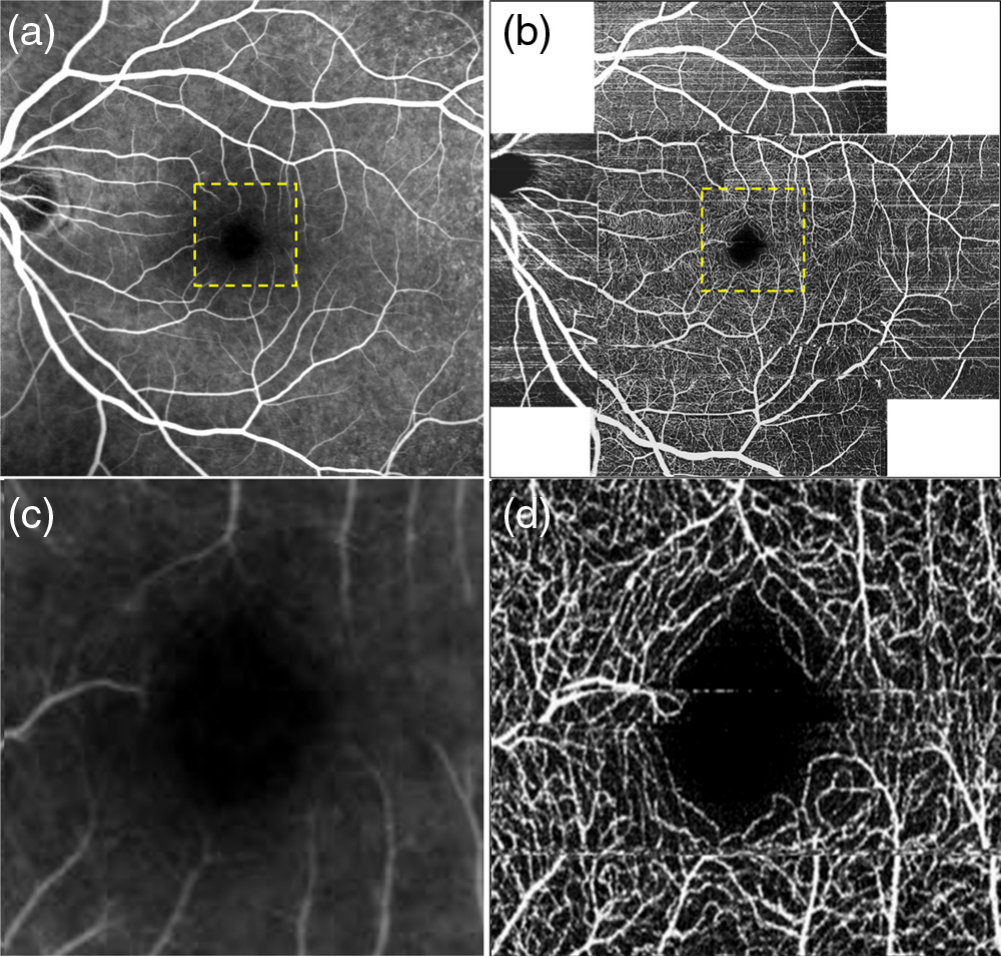
Comparison of FA, 3 min after fluorescein injection (a, c) with PV OCTA *en face* projection of the retinal vasculature [(b and d) 30-deg mosaic composed of four 5-mm×5-mm OCT images]. Right row: zoom at 1.5-mm×1.5-mm area centered at the fovea. Image sizes: (a and b) 9  mm×9  mm and (c and d) 1.5  mm×1.5  mm.

**Fig. 11:**
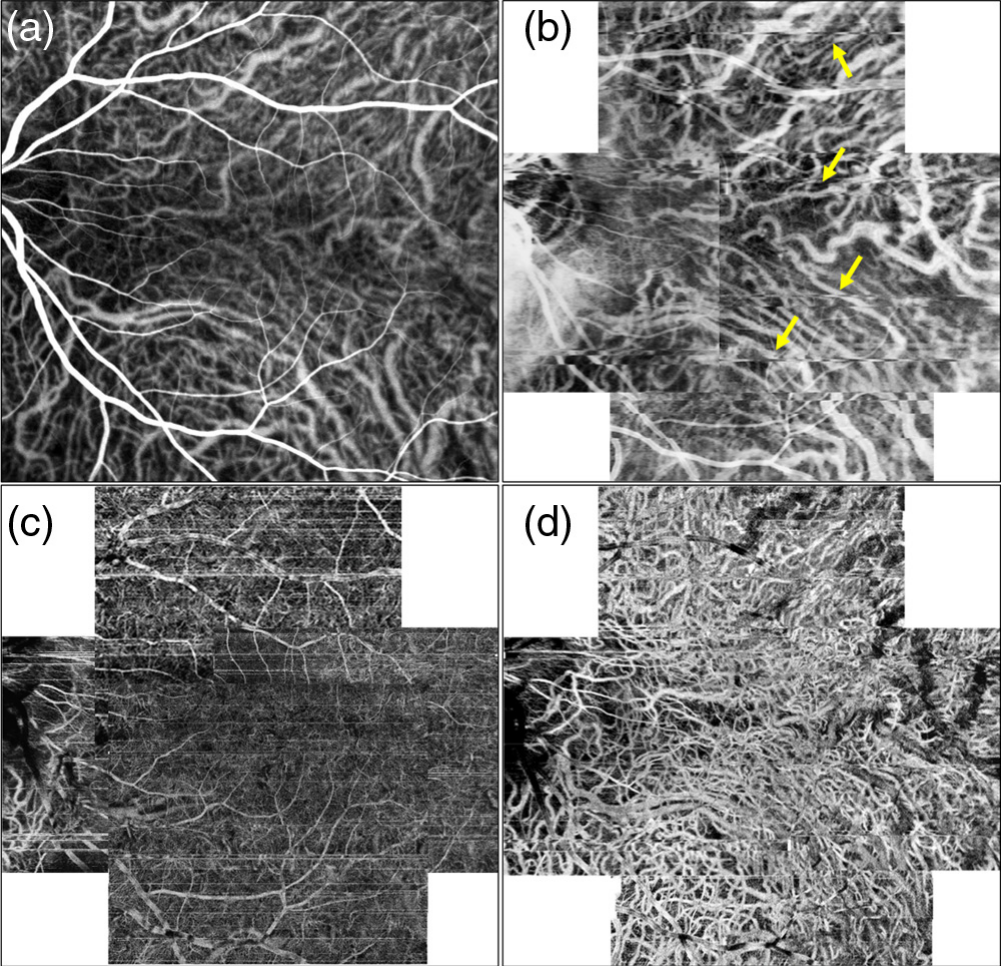
Comparison of ICG angiography, (a) 30 s after the fluorophore injection with (b) OCT intensity projection of the choroid and(c) PV OCTA *en face* projection of the choriocapillaris, and (d) deeper choroid. OCT projections are mosaic composed of four 5-mm×5-mm OCT images spanning 30-deg FOV.

In Figs. 9–11, we demonstrate the results of stitching images from 4 3-D datasets, each covering an area of 5×5  mm of the retina. The resultant mosaics span 30-deg FOV, enabling side-by-side comparison with corresponding images obtained using standard ophthalmic diagnostic methods.

Figure 9 compares a color fundus photograph with an OCT intensity projection image obtained from the healthy volunteer (Caucasian female, age: 51 years). The mosaic generated from full retinal depth projections [Fig. 9(b)] shows a network of large vessels, with structural details similar to FP [Fig. 9(a)]. However, it lacks the color discrimination of features provided by the fundus photos, which enables differentiation between large arteries and veins. Although this OCT image was generated from data with B-scan repeats to enable OCT angiography computations, data acquisition replications are not required in the OCT intensity projection imaging.^37^ Capturing OCT intensity data spanning a 30-deg FOV, without exceeding the half-of-the-$1/e^{2}$ beam spot diameter step, would take only 1 s with 1.7-MHz imaging rate. Imaging a 60-deg FOV would take 3.8 s. However, only limited information about retinal vascular structures is available, e.g., retinal capillaries are not visualized in full depth intensity projections, and can be visualized only in projections from segmented selected retinal layers.^37^^,^^39^ To reiterate, 1.7-MHz imaging rate could be utilized for high-definition (densely sampled), wide field, intensity OCT imaging in single datasets but only at the cost of losing vascular imaging capability and imaging sensitivity.

Figure 10 enables comparison of retinal vascular imaging with FA and OCT angiography at 1.7-MHz A-scan rate. OCT angiography has an advantage over the FA in imaging of the architecture of capillary vessels. Selected regions of interest of the 30-deg FOV mosaic obtained from the FDML SSOCT system can be enlarged to analyze the details of retinal capillaries [Figs. 10(c) and 10(d)], which are not visible in FA. However, FA can provide information about time dynamics of blood distribution in different vessels, which is not available with OCTA. Also, OCTA imaging relies on the motion contrast, i.e., OCTA signal can be detected from the flow of highly scattering blood cells, which is sufficiently fast to introduce measurable changes of the OCT signal. In consequence, leakage of blood or plasma from the vessels is not directly visualized in OCTA images.

Perhaps the most striking results were obtained in the imaging of the choroid (Fig. 11). The OCT intensity projection displayed in an inverted gray scale [Fig. 11(b)] mimics remarkably well the appearance of large choroidal vessels in ICGA 30 s after the injection of the fluorophore. The main difference between OCT angiography at 1.7-MHz A-scan rate and standard clinical diagnostic methods can be assessed from Figs. 11(a), 11(c), and 11(d). The 30-deg FOV OCTA mosaics reveal features not visualized in the ICG angiography. The ICGA not only misses the meshwork of capillary vessels [Fig. 11(c)] but also has limited capability to visualize an extremely rich network of highly convoluted medium-sized vessels [Fig. 11(d)]. Since the OCTA mosaics were generated from datasets acquired with high scanning density, regions of interest can be enlarged at any location of the 30-deg FOV to reveal highly detailed vascular features. The similarity between ICGA and OCTA is in the intrusion of retinal vessels in visualization of the choroid. In ICGA, the visibility of retinal vessels is caused by filling of the retinal vasculature with the fluorophore. In OCTA, the visibility of retinal vasculature in the projections of the choroid is caused by retinal blood flow projections.^36^

Eye motion is one of the main challenges in wide-field OCTA imaging. The imaging time required to collect each of the datasets covering 5  mm×5  mm area of the eye fundus with the FDML SSOCT system was 6 s. Although there is no apparent degradation of the image quality related to possible drying of the tear film during the extended imaging time, motion artifacts are visible in all images. Artifacts connected to involuntary saccadic motion of the eye manifest as discontinuities in vessels, often accompanied by horizontal lines of high OCT angiography signal intensity [examples indicated by arrows in Fig. 10(b)].

### 3.3 Demonstration of 1.7-MHz A-Scan Rate OCT Angiography Imaging in Advanced AMD

**Fig. 12:**
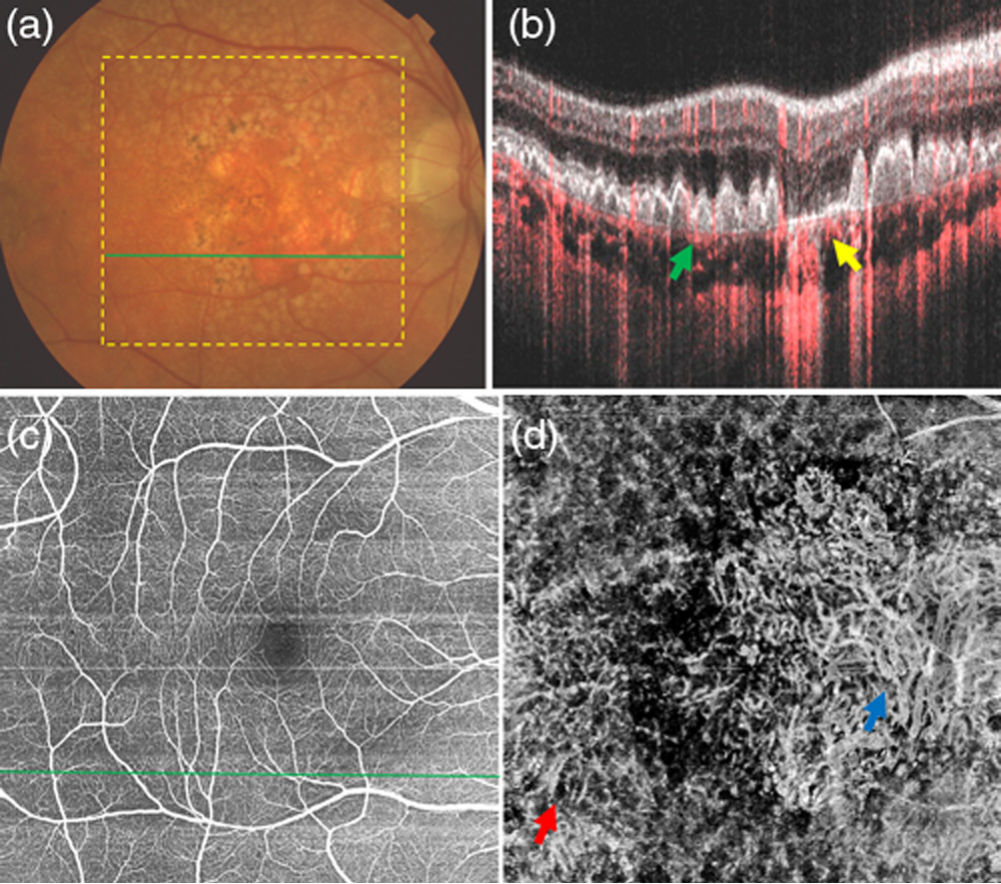
Patient P1, 60-years Caucasian male diagnosed with geographic atrophy. (a) Fundus photograph and (b) OCT (gray scale) and OCTA (red scale) composite B-scan image; green arrow indicates large drusen, yellow arrow points at the RPE atrophy. (c) *En face* projection of the retinal vasculature. (d) OCTA projection of combined Sattler’s layer and Haller’s layer vessels; blue arrow indicates vessels in GA area, red arrow points vessels outside GA area. Green line in (a) and *en face* angiograms (c) showing the location of the B-scans (b). OCT image sizes: 9  mm×9  mm (30-deg FOV).

**Fig. 13:**
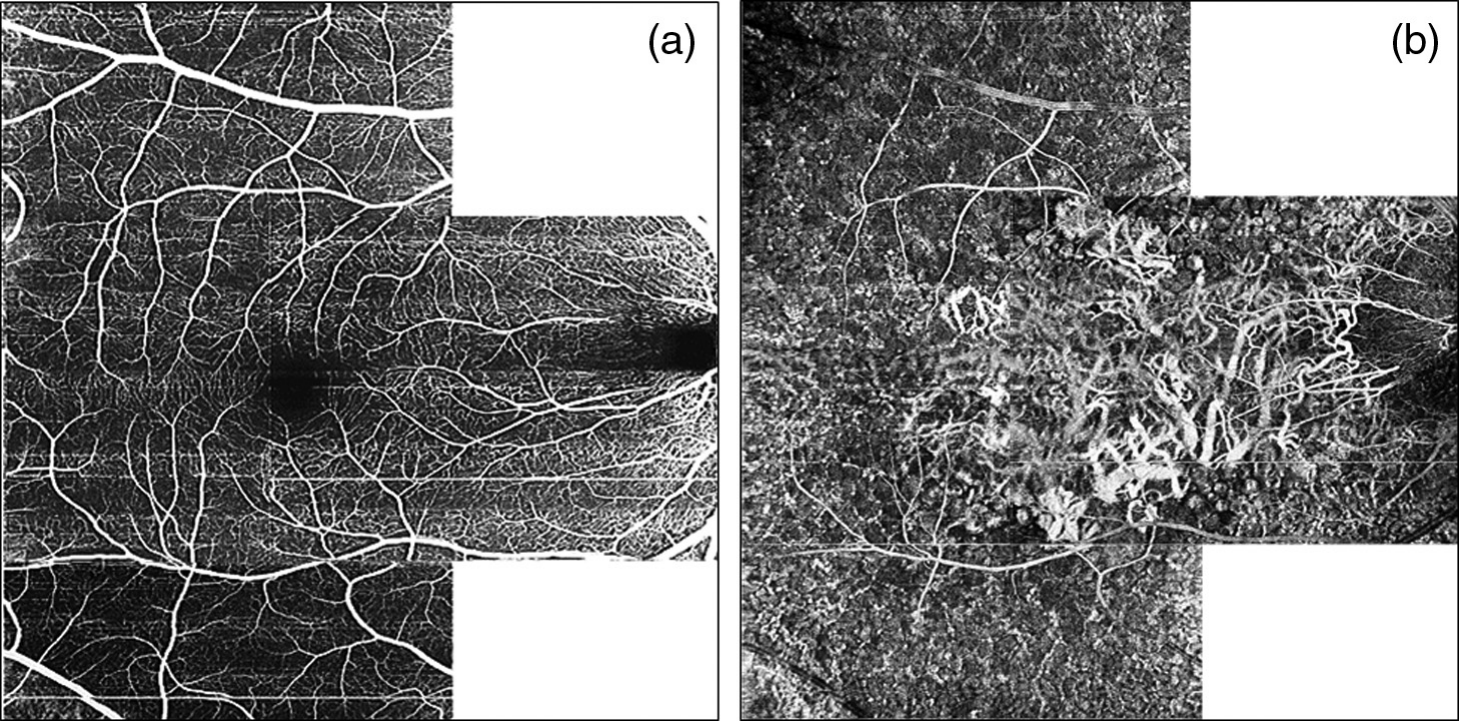
Patient P1, 60-year-old Caucasian male, PV OCTA *en face* projections of (a) the retinal and (b) choroidal vasculature. Mosaics were generated from three datasets covering 5  mm×5  mm, each. Image sizes: ∼9  mm×6  mm (25-×18-deg FOV).

**Fig. 14:**
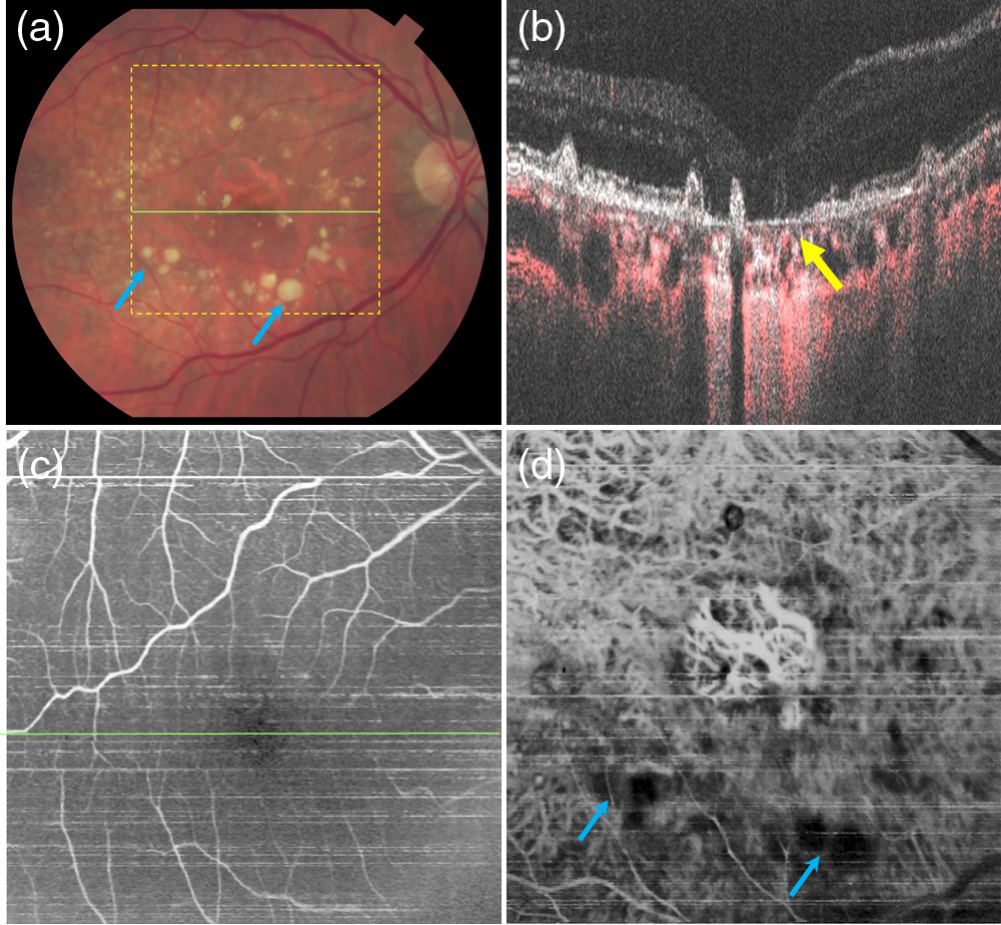
Patient P2, 67-year-old Caucasian female, exudative AMD treated with anti-VEGF injections. (a) Color fundus photograph (35-deg FOV). (b) OCT (gray scale) and OCTA (red scale) composite B-scan image with yellow arrow points at the RPE atrophy. (c) *en face* projection view of retinal layers. (d) OCTA projections of combined Sattler’s layer and Haller’s layer vessels, blue arrows point at the low signal areas corresponding to drusen in (a). Green line in (a) and *en face* angiograms (c) showing the location of the B-scans (b). OCT image sizes: 5×5  mm (16-deg FOV).

**Fig. 15:**
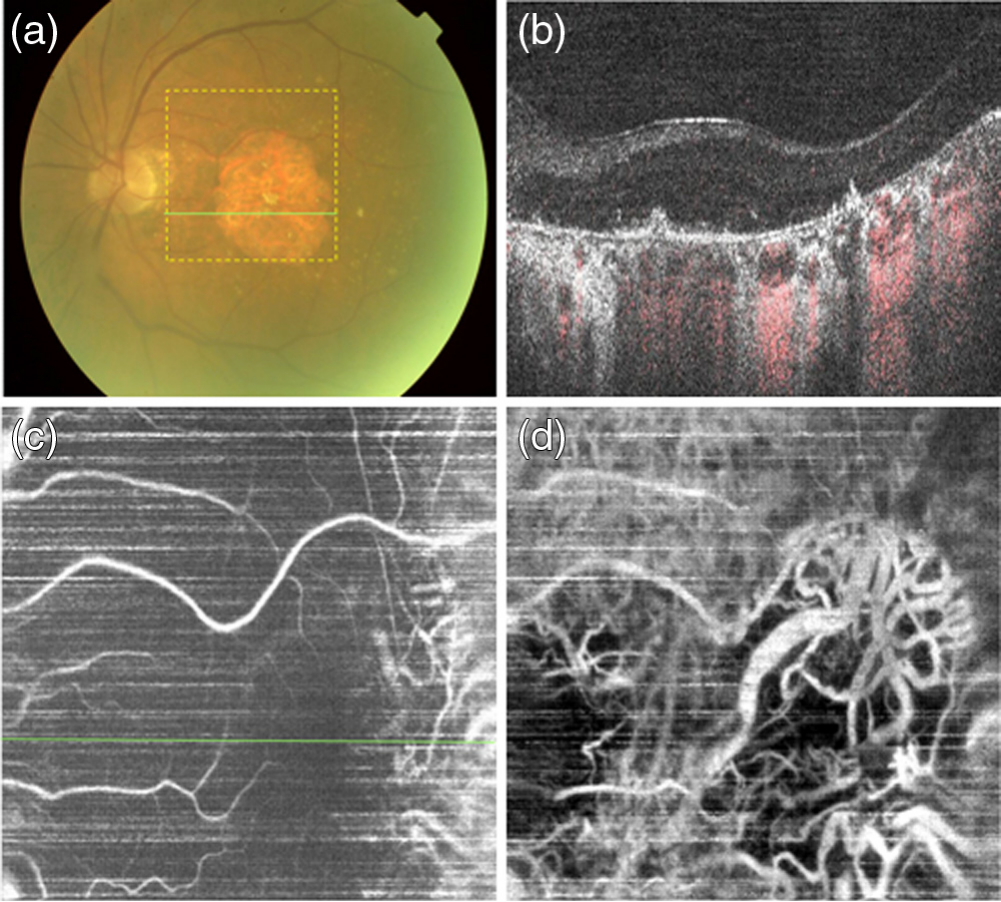
Patient P3, 81-year-old Caucasian male with nonexudative AMD, with geographic atrophy and cataract. (a) Color fundus photograph, 35-deg FOV. (b) OCT (gray scale) and OCTA (red scale) composite B-scan. (c) *En face* projection view of retinal layers. (d) OCTA projections of combined Sattler’s layer and Haller’s layer vessels. Green line in (a) and *en face* angiograms (c) showing the location of the B-scans (b). OCT image sizes: 5  mm×5  mm (16-deg FOV).

Imaging of eyes with developing pathology provides additional challenges in comparison with imaging of normal subjects. Figures 12–15 show three cases of advanced AMD. Patient P1, age 60 years, Caucasian male (Fig. 12), was diagnosed with extensive large drusen [green arrow in Fig. 12(b)], central GA, and juxtafoveal lobules of GA (yellow arrow). He had good transparency of ocular media and good eye stability, which enabled imaging of the retinal and choroidal vasculature with contrast similar to that of a normal eye. A rich network of fine retinal capillary vessels is visible throughout the entire 30-deg FOV [Fig. 12(c)], with the 8-μm scan step. In the OCTA *en face* projection of the choroid, a dense network of normal appearing large choroidal vessels is visible in the RPE atrophy areas. Choroidal vessels were not visualized outside the GA with an exception of sparse areas where their visibility is poor [red arrow in Fig. 12(d)]. This is most likely due to the presence of large confluent drusen, covering extensive areas at the border and outside the GA, and causing attenuation of light penetrating toward the choroid. Similar effect of low OCTA signal from the choroid, coinciding with drusen can be observed in Fig. 14(d). For comparison, a 25-deg FOV mosaic composed of three 5  mm×5  mm PV OCTA *en face* projections is presented in Fig. 13. Although the scan step was smaller (5  μm), no new findings were revealed in comparison to Fig. 12. The retinal vasculature [Fig. 12(a)] shows a similarly detailed network of vessels down to the capillary level. The choroidal vasculature has a similar appearance to Fig. 12(d). This observation may suggest that in advanced stages of disease development, with large-scale pathological changes, the advantages of a large FOV may surpass the need for high detail imaging.

Patient P2 (age 67 years, Caucasian female) had exudative macular degeneration treated with anti-vascular endothelial growth factor (anti-VEGF) injections (4 times) (Fig. 14). The area of RPE damage was smaller than in patient P1 with GA. However, the OCT imaging sensitivity was lower than in P1, most likely due to poorer transparency of the ocular media, resulting in low visibility of the inner retinal layers [Fig. 14(b)]. In addition, due to lower eye stability during the imaging, motion artifacts have higher influence on the image quality than in P1.

The OCTA *en face* projection of the choroid [Fig. 14(d)] visualizes large vessels throughout the entire image. However, the vessels are less sharply defined than in a normal eye except in the area of GA, where clear visualization of large vessels was achieved. In addition, focal areas of low OCTA signal are visible in the choroidal *en face* projection, which coincides with the location of drusen in the fundus photograph [arrows in Figs. 14(a) and 14(d)].

Patient P3, an 81-year-old Caucasian male with nonexudative AMD, with geographic atrophy and cataract, had the most advanced pathology of the presented cases. A very extensive GA area spanned the majority of the macular region of the retina. Poor transparency of the ocular media degraded the sensitivity of OCT imaging resulting in poor visualization of the inner retinal layers [Fig. 15(b)] and, in consequence, also in poor visualization of the retinal vasculature in the OCTA *en face* projection [Fig. 15(c)]. The choroidal vessels are visible in the entire imaged area [Fig. 15(d)]; however, their appearance is diffuse outside the GA areas. In the GA areas, the vessels are sharply defined but notably, their density appears to be much lower than in the GA area in patient P1 [Fig. 12(d)] and in the normal eye [Figs. 7 and 11(c)]. This may suggest loss of deeper vessels due to the development of pathology.

The choriocapillaris layer was not visualized in any of the *en face* projections of the three presented patients. Two possible explanations are choriocapillaris atrophy, and errors in image segmentation and flattening. The composite OCT–OCTA cross-sectional images reveal only sparse spots of OCTA signal within a narrow layer abutting the Bruch’s membrane in P1 [Fig. 12(b)], whereas a corresponding layer of the normal eye is more densely occupied by choriocapillaris [Fig. 12(b)]. In patients P2 and P3, choriocapillaris is nearly absent in the cross-sectional images [Figs. 14(b) and 15(b)]. This observation may support the hypothesis of choriocapillaris atrophy. However, the image segmentation and flattening errors cannot be ruled out either. Geographic atrophy is perhaps one of the most challenging pathologies for data processing algorithms aiming at segmentation of the RPE. Not only is the RPE missing in large areas of the retina but also large drusen disrupting the laminar appearance of the outer retinal layers are often present adjacent to GA. As a result, data segmentation algorithms often fail in correct identification of the RPE location and in approximation of the normal curvature of the outer retina, which serves as a reference for the *en face* projection image generation. Segmentation errors are present in all presented cases affecting the visualization of choriocapillaris and deeper choroid. Image flattening errors are visible in the right bottom corner of Fig. 14(c) (diminished visibility of retinal vessels) and in the right edge of Fig. 15(c) (intrusion of the choroidal vasculature in the retinal *en face* projection image visible as a band of high OCTA signal).

## 4 Discussion and Conclusions

In this study, we have used an FDML SSOCT system with imaging speed of 1.7 million A-scans/s to demonstrate advantages and challenges of “wide-field” OCTA imaging of the posterior segment of the human eye. In comparison with previous reports on wide-field OCTA imaging (Table 6), we have implemented scan protocols with equal scanning density in the horizontal and vertical directions, with scan steps of ∼0.4 and ∼0.6 of the $1/e^{2}$ beam spot diameter at the retina (5 and 8  μm, respectively). We have demonstrated that scanning steps smaller than half of the $1/e^{2}$ beam spot diameter enable visualization of vascular features down to the capillary level and are important in visualization of choriocapillaris layer morphology. However, they limit the FOV of the data acquired within a time limit of 6 s to 16 deg (5  mm×5  mm). Imaging with scan steps exceeding half of $1/e^{2}$ beam spot diameter (8  μm) enabled widening the FOV captured in a single dataset to 30 deg. Good rendering of retinal capillaries was still achieved at this scanning density but visualization of choriocapillaris architecture had lower apparent contrast. In comparison, slower imaging systems (∼70 to 100 kHz) undersample the scanned area (scan step larger than the $1/e^{2}$ beam spot diameter), yet still cannot achieve the standard ∼35-deg FOV offered by the classic fundus angiography (Table 6). We demonstrated that in a commercial OCT system operating at a 68-kHz A-scan rate, with 10-deg FOV, scanning steps comparable to the beam spot diameter provide details of the retinal vasculature similar to features visualized with the FDML SSOCT system at 1.7-MHz imaging rate with 30-deg FOV. However, undersampling (17-μm scanning step, 20-deg FOV) resulted in loss of information about the retinal capillary network. This observation is consistent with a previous report on wide-field FDML SSOCTA imaging at 1.7-MHz A-scan rate^38^ in which 48-deg FOV, i.e., nearly the FOV of a classic wide-field FA, was achieved by exceeding the half beam spot diameter step size in the fast scan axis, and undersampling in the slow scan axis (Table 6). The authors reported visualization of large retinal vessels but did not achieve visualization of the retinal capillaries nor choriocapillaris morphology. Only narrowing the FOV to 12 deg revealed the retinal capillary system and “gaps” (flow voids) in the OCTA signal from the choroid, characteristic of the choriocapillaris layer.

**Table 6:** Comparison between different OCT angiography systems.

	Current paper		Blatter et al.^38^	Wang et al.^44^	Angiovue™	Zeiss AngioPlex^45^^,^^48^
					Optovue^43^	
Parameters						
A-scan rate	1.7 MHz	1.7 MHz	1.7 MHz	100 kHz	70 kHz	68 kHz
Scanning area	$5\times 5\;{\mathrm{mm}}^{2}$	$9\times 9\;{\mathrm{mm}}^{2}$	$16\times 16\;{\mathrm{mm}}^{2}$	$9\times 9\;{\mathrm{mm}}^{2}$, $12\times 12\;{\mathrm{mm}}^{2}$	$6\times 6\;{\mathrm{mm}}^{2}$, $8\times 8\;{\mathrm{mm}}^{2}$	$6\times 6\;{\mathrm{mm}}^{2}$
B-scan repeats	5	5	5	2	2	2
Number of B-scans	1024	1152	800	500	304	350
Number of A-scans per B-scan	1024	1152	2060	500	304	350
Beam spot diameter (μm)	14	14	14	15	15	15
Horizontal step size	5 μm	8 μm	8 μm	18 μm for $9\times 9\;{\mathrm{mm}}^{2}$	20 μm for $6\times 6\;{\mathrm{mm}}^{2}$	17 μm
Vertical step size	5 μm	8 μm	20 μm	18 μm for $9\times 9\;{\mathrm{mm}}^{2}$	20 μm for $6\times 6\;{\mathrm{mm}}^{2}$	17 μm
B-scan acquisition time (ms)	1.2	1.35	1.12	5	5	5
Volume acquisition time (s)	6	8	7	5	5	5
Line scan ophthalmoscope or eye tracking	No	No	No	Yes	Yes	Yes

OCT angiography has been shown to provide detailed, depth-resolved images of vascular structures of the retina and choroid down to the capillary level. However, the challenge is in achieving a large FOV within imaging times not exceeding 5 to 10 s. A common solution to enable wide FOV without sacrificing the visualization of fine vascular features is implementation of mosaic-like imaging of the eye fundus. We have demonstrated that with 1.7-MHz imaging rate a 30-deg FOV can be visualized with only four datasets acquired at neighboring locations in the retina. We have compared the OCTA *en face* projections with “standard FOV” (∼35 to 40 deg) ophthalmic imaging. The OCT counterpart of FP is intensity (or structural) OCT imaging. It has the potential to achieve wide FOV as defined by clinical diagnostic standards, since it does not require repetitive acquisition of data from the same location in the eye fundus, essential in OCT angiography. However, only limited information about the vascular structures of the eye can be obtained from structural OCT imaging and no color information is available. Information corresponding to FA and ICGA can be obtained from OCT angiography imaging. OCTA can provide images of fine retinal vascular structures typically not observed in FA imaging. OCT imaging of the retinal vascular architecture was available since the advent of the OCTA techniques. However, only high-speed systems at ∼1050-nm wavelength imaging regimes are currently capable of visualization of the choroidal blood flow. We have demonstrated unprecedented detail and richness of choroidal vascular architecture imaged in a normal human eye imaged at 1.7-MHz A-scan rate.

In addition, comparison between the OCT and classic angiographic imaging of the eye highlights the speed challenge in wide-field OCTA, not present in FP and angiography. Acquisition of OCTA datasets enabling visualization of retinal capillaries and choriocapillaris throughout a modest 16-deg FOV takes 6 s at 1.7-MHz imaging rate. During that time, involuntary eye motion may introduce artifacts obscuring the images of the posterior eye segment vasculature, including discontinuities of vessels caused by saccadic motion and distortions of the vascular networks caused by eye drifts. The severity of image deformation depends on the individual subject’s eye stability during the OCT scanning. As demonstrated in the imaging of geographic atrophy cases, degradation of image quality caused by motion artifacts can be negligible (patient P1) or may substantially degrade the quality of images (patient P3). This observation indicates the need for development of experimental and numerical motion artifact compensation methods for OCT angiography, especially when wide-field imaging is considered.

Imaging of geographic atrophy cases revealed yet another challenge associated with depth-resolved OCTA visualization of vascular layers. With increasing severity of outer retinal pathology disrupting the laminar arrangement of the retinal layers and the integrity of the RPE, the numerical image segmentation and flattening methods are more susceptible to errors. Such errors may cause generation of incomplete or fragmented *en face* projection images of vascular networks, especially confined to narrow depth ranges (e.g., choriocapillaris). Caution is advised when interpreting the OCTA projections if there is a suspicion of failure in the image segmentation algorithm.

As a final remark, we address a common misconception connected to high-speed OCT angiography imaging. Increasing the imaging speed in OCT systems with beam scanning does not allow imaging time to be shortened to the point that no eye motion occurs during the data acquisition. Aside from the consideration of the imaging sensitivity diminishing with increasing imaging speed, the dynamics of the blood flow needs to be taken into account when developing experimental OCTA imaging protocols. OCT angiography relies on the detection of signal fluctuations (variation of magnitude and/or phase of the complex amplitudes) originating from moving blood cells. Therefore, the same locations of the eye fundus must be imaged repeatedly over a time period sufficiently long to enable observable signal change (i.e., change of the magnitude and/or phase of the complex amplitude larger than amplitude and/or phase noise levels). The length of this time period depends on the blood flow velocity in the vascular systems intended for visualization in OCTA angiograms. The slower the flow, the longer the time period of the repeated scanning required to detect signal change caused by moving blood cells and to visualize the location of vessels, at given imaging sensitivity and noise levels. Thus, the time limits in OCT angiography imaging are set not only by the speed of state-of-the-art OCT systems, but also by the dynamics of the ocular blood circulation.
